# Solid state fermentation of *Moringa oleifera* leaf meal by mixed strains for the protein enrichment and the improvement of nutritional value

**DOI:** 10.7717/peerj.10358

**Published:** 2020-11-18

**Authors:** Honghui Shi, Bin Su, Xiaoyang Chen, Ruiqi Pian

**Affiliations:** 1 College of Forestry and Landscape Architecture, South China Agricultural University, Guangzhou, Guangdong, China; 2 Guangdong Province Research Center of Woody Forage Engineering Technology, Guangzhou, Guangdong, China; 3 Guangdong Research and Development Centre of Modern Agriculture (Woody Forage) Industrial Technology, Guangzhou, Guangdong, China; 4 Guangdong Key Laboratory for Innovative Development and Utilization of Forest Plant Germplasm, Guangzhou, Guangdong, China; 5 State Key Laboratory for Conservation and Utilization of Subtropical Agro-bioresources, Guangzhou, Guangdong, China

**Keywords:** Solid-state fermentation, *Moringa oleifera* leaf meal, True protein, Response surface methodology, Nutritional value

## Abstract

*Moringa oleifera* Lam. (MO) is a fast-growing multi-purpose deciduous tree with high biomass and nutritional value. However, the presence of antinutritional factors, poor palatability, and indigestibility of *Moringa oleifera* leaf meal (MOLM) restrict its application to animal feed. This study aimed to obtain high-quality protein feeds via solid-state fermentation (SSF) of MOLM. The process conditions for increasing the true protein (TP) content using *Aspergillus niger*, *Candida utilis* and *Bacillus subtilis* co-cultures were optimized, and the chemical composition of MOLM was compared before and after fermentation. The results of this study showed that the highest TP content could be obtained through mixed-strain culture of *A. niger*, *C. utilis* and *B. subtilis* at a ratio of 1:1:2. The MOLM was inoculated with *A. niger*, followed by *C. utilis* and *B. subtilis* 24 h later. The optimized co-culture parameters were as follows: total inoculation size, 24%; temperature, 32 °C; fermentation time, 6.5 days; and initial water content, 60%. The maximum TP yield was 28.37%. Notably, in the fermented MOLM (FMOLM), the content of nutrients such as crude protein (CP), small peptides, and total amino acids (AAs) were significantly increased relative to unfermented MOLM, whereas the contents of crude fiber (CF), tannin, and phytic acid were significantly decreased. MOLM analysis using scanning electron microscopy (SEM) revealed that SSF disrupted the surface structure of MOLM, and sodium dodecyl sulfate–polyacrylamide gel electrophoresis (SDS-PAGE) indicated that macromolecular proteins were degraded. The in vitro protein digestibility (IVPD) of FMOLM was also improved significantly. Our findings suggest that multi-strain fermentation with *A. niger*, *C. utilis* and *B. subtilis* improves the nutritional quality of MOLM, rendering it a viable functional feedstuff for use in livestock industries in the future.

## Introduction

The world population is expected to reach 9.7 billion by 2050 (UN, 2017), and the global demand for meat and milk is projected to increase by 57% and 48%, respectively (Alexandratos & Bruinsma, 2012). As a result, livestock production is predicted to increase by 21% between 2010 and 2025 (Mottet et al., 2017). Current feedstocks include cereals that are suitable for human consumption. Up to 50% of global cereal is expected to be used as feed instead of human food by 2050 (Legg, 2017). The global demand for animal protein for human nutrition is continuously rising, leading to an increase in the cost of livestock feed concentrates (Tufarelli, Ragni & Laudadio, 2018). To meet the increasing demand for animal products, an alternative source of feed not required as a human food must be developed.

Trees and other browse species have been used as livestock fodder for centuries. Due to the rising price of soybean meal, woody forage is expected to become a common source of high-quality vegetable protein for animal rations. *Moringa oleifera* Lam. (MO) is a multi-functional fodder tree with desirable characteristics such as high yield, good adaptability, simple culturing methods, and strong resistance to environmental stressors. Moreover, MO has high nutritional value, containing important nutrients such as proteins, vitamins, minerals and phytochemicals (Elghandour et al., 2017; Leone et al., 2015; Raman, Alves & Gnansounou, 2018). For a review of the nutrient composition of *Moringa oleifera* leaf meal (MOLM), see Falowo et al. (2018). The crude protein (CP) content of MOLM ranges between 10.7–30.3% dry matter (DM), while that of crude fiber (CF) is between 7.1–35.0%. The unsaturated fatty acid content of MOLM is typically higher than that of saturated fatty acids; MOLM contains 14–17 fatty acids, of which *α*-linolenic acid constitutes 57% of the total (Moyo et al., 2011). MO also contains a large number of bioactive compounds (Maizuwo et al., 2017); the leaves of MO, which are the most used part of the plant, are rich in flavonoids, alkaloids, polyphenols and polysaccharides. The anti-oxidative, anti-inflammatory, anti-microbial, and “folk medicinal” properties of MOLM can be attributed to the presence of functional phytochemicals, such as isothiocyanate, pterygospermin, chlorogenic acid, kaempferol and zeatin (Tumer et al., 2015). This combination of properties provides MOLM with great potential for becoming a novel livestock feed resource.

In a recent study, alfalfa meal was replaced with MO leaves as feed for rabbits, which had positive effects on rabbit growth performance, meat quality, antioxidant levels and other biochemical parameters (Sun et al., 2018). Sun et al. (2020) also recently reported that MO supplementation could enhance reproduction performance, elevate protein levels in the colostrum, and improve serum antioxidant levels in both sows and piglets. Furthermore, supplementation with MO may contribute to improved immunity, health, and reproductive performance in poultry (Mahfuz & Piao, 2019).

Despite the clear benefits of MOLM supplementation, its use is limited due to the presence of antinutritional factors, poor palatability, and indigestibility. The antinutritional factors, though not toxic or fatal, could potentially interfere with the digestion and absorption of other important nutrients such as zinc, iron, calcium (Ca), and magnesium when consumed in large quantities (Makkar, Francis & Becker, 2007; Nouman et al., 2014). A process for improving MOLM fodder quality is therefore imperative.

Solid-state fermentation (SSF) is a fermentation process that takes place on a solid support in the near-absence of free water, and is a cost-effective method of bioproduct synthesis (Salgado et al., 2015). SSF systems have been effectively applied to MOLM to increase its protein content, while reducing undesirable substances such as fiber, tannin, and phytic acid (Thierry et al., 2013; Zhang et al., 2017). In 2013, the commonly used fermentation microorganisms *Aspergillus niger*, *Bacillus subtilis*, and *Candida utilis* were granted generally regarded as safe (GRAS) status in China, and can thus be safely and legally added to animal feed.

In this study, the SSF conditions for *A. niger*, *B. subtilis* and *C. utilis* were optimized using response surface methodology (RSM). The chemical composition and *in vitro* protein digestibility (IVPD) of FMOLM were also assessed under optimal fermentation conditions. To further understand the effects of SSF on the physicochemical properties of FMOLM, the microstructure of MOLM before and after fermentation was observed by scanning electron microscopy (SEM). Changes in protein molecular weight during fermentation were also evaluated by SDS-PAGE.

## Materials and Methods

### Materials

MOLM was provided by an MO-planting professional cooperative in Xuwen County, Zhanjiang City, Guangdong Province, China. MOLM was ground until small enough to pass through a 40-mesh sieve and then stored at room temperature. *A. niger* (GIM 3.576) and *B. subtilis* (GIM 1.427) were obtained from the Guangdong Culture Collection Center. *C. utilis* (CICC 31188) was purchased from the China Centre of Industrial Culture Collection (Beijing, China).

### Inoculum preparation

For inoculum preparation, *A. niger* was grown on potato dextrose agar (PDA) slants at 30 °C for 7 days; spores were harvested by rinsing the slants with sterile water containing 0.01% Tween-80, achieving spore suspensions of around 10^7^ spores/ mL. *B. subtilis* was cultured in de Man Rogosa Sharpe (MRS) medium (Huankai Microbial Sci. & Tech Co., Ltd., Guangzhou, China) at 28 °C for 2 days. *C. utilis* was cultivated in yeast extract agar medium (20 g/ L glucose, 20 g/ L peptone, 10 g/ L yeast extract, pH 7.2–7.4) at 30 °C for 120 h. Liquid seed cultures for *B. subtilis* and *C. utilis* contained their respective cultivation media without agar. Culture suspensions were adjusted to 10^8^ CFU/ mL with sterilized physiological saline solution.

### Single factor experimental method

MOLM was sterilized at 121 °C for 20 min and cooled to room temperature. Factors that can influence the true protein (TP) content of MOLM under SSF, including the inoculation ratio of *A. niger*, *C. utilis* and *B. subtilis*, inoculation order and size, fermentation temperature and duration, and initial moisture content were optimized by a technique that varies each parameter in a stepwise manner.

Experiments were performed in 500 mL Erlenmeyer flasks containing 40 g MOLM. *A. niger*, *C. utilis,* and *B. subtilis* were inoculated to MOLM for SSF in four ratios (1:1:1, 1:2:1, 1:1:2, 2:1:1). Fermentation was performed using different inoculation orders (listed in Table 1) and fermentation durations (6, 7, 8, 9, 10 days). The effects of inoculation size (8%, 12%, 16%, 20, 24%), fermentation temperature (24, 27, 30, 33, 36 °C) and initial moisture content (30%, 40%, 50%, 60%, 70%) were also analyzed for SSF optimization. SSF treatments were carried out in two sets, with three biological replicates.

**Table 1:** Inoculation orders of MOLM.

Treatments	Inoculation interval time (h)		
	*A. niger*	*C. utilis*	*B. subtilis*
T1	0	0	0
T2	0	0	24
T3	0	0	48
T4	0	24	0
T5	0	24	24
T6	0	24	48
T7	0	48	0
T8	0	48	24
T9	0	48	48

### Experimental design for response surface methodology (RSM)

RSM was performed to evaluate the influence of inoculation size, fermentation temperature, and fermentation time on TP content. Table 2 shows the independent variables in the Box-Behnken design (BBD). A total of 17 experimental runs were performed to optimize the three independent variables. Each run was carried out in triplicate. The mean TP content of FMOLM after each experimental run was used in the RSM calculations.

**Table 2:** Coded and uncoded levels of independent variables used for Box-Behnken design.

Uncoded level	Coded level		
	−1	0	1
Inoculation sizes (A, %)	8	16	24
Fermentation temperature (B, °C)	27	32	37
Fermentation time (C,h)	5	7	9

### Chemical analysis

After fermentation, FMOLM samples were dried at 50 ° C for 12 h, ground and passed through a 40-mesh sieve. Proteins were precipitated from the FMOLM with trichloroacetic acid, and TP was measured using the Kjeldahl method (Licitra, Hernandez & Van Soest, 1996) and the Foss Kjeltec 2200 Auto Distillation Unit (FOSS, Hillerød, Denmark).

MOLM and FMOLM samples were analyzed for their DM, CP, ether extract (EE), CF, neutral detergent fiber (NDF), acid detergent fiber (ADF), acid detergent lignin (ADL), ash, Ca and total phosphorus (P) contents according to (AOAC, 2005). Hemicellulose content was calculated as the difference between NDF and ADF, while cellulose content was calculated as the difference between ADF and ADL. The total sugar content was estimated using the phenol-sulfuric acid method (Dubois et al., 1951). The L-8900 instrument (Hitachi, Tokyo, Japan) was used for analyzing the amino acid (AA) profiles of uninoculated and inoculated MO leaves. Samples were hydrolyzed with 6 M HCl at 100 °C for 24 h under a vacuum prior to AA profile analysis.

The small peptide content was calculated by subtracting free amino acids (FAAs) from trichloroacetic acid-soluble protein (TCP-SP). The TCP-SP was analyzed as reported by Ovissipour et al. (2009). FAAs were extracted from MOLM and FMOLM with 0.02 M HCl and measured using the L-8900 instrument.

### Analysis of anti-nutritional factors

Total phenols and tannins were determined according to a previously published method using tannic acid as a standard (Makkar et al., 1993). Total phenolics were measured using Folin-Ciocalteu reagent. Tannins were quantified in FMOLM extracts by calculating the difference in total phenolics before and after tannin removal using insoluble polyvinylpyrrolidone.

Phytic acid content was analyzed using the spectrophotometric method described by Gao et al. (2007): ground samples (0.5 g each) were stirred for 1 h in 100 mL of 2.4% HCl at room temperature, and then decanted and filtered. A 5 mL sample of the filtrate was diluted with 25 mL of distilled water, and 15 mL of 0.7 M sodium chloride was added. Absorbance was measured at 520 nm using a UV-visible spectrophotometer (CE 2041; Cecil Instruments, Cambridge, UK).

### In vitro protein digestibility (IVPD)

In vitro two-stage enzyme hydrolysis was carried out according to a previously published method (Boisen & Ferna, 1995). In brief, 1 g samples were transferred to 100 mL conical flasks containing 25 mL phosphate buffer (0.1 M, pH 6.0). pH was adjusted to 2.0 with 1 M HCl and 1 mL 10,000 U/ mL pepsin (activity: 3,000 U/ mg; Sigma, St. Louis, MO, USA) was blended evenly into the suspension, followed by incubation at 39 °C with 150 rpm agitation for 6 h. Then, 10 mL 0.2 M phosphate buffer (pH 6.8) and five mL 0.6 M NaOH were added to the suspension, and the pH was adjusted to 6.8 with 1 M NaOH solution. Then, 1 ml 625 U/ mL trypsin (activity: 250 U/ mg; Sigma) was added, and the suspension was incubated at 39 °C with 150 rpm shaking for 18 h. After completion of digestion, 5 ml 20% sulfosalicylic acid was added to precipitate the proteins. The supernatants were discarded and the undigested residue was dried at 80 °C overnight. The IVPD was calculated based on the difference in nitrogen content between the original sample and the undigested residue, following correction for nitrogen detected in the blank.

### Scanning electron microscopy (SEM)

Samples for SEM were mounted onto an aluminum stub and coated with gold. Specimens were viewed with a field-emission scanning electron microscope (JSM-7500F; JEOL, Tokyo, Japan) at 400×, 1,000× and 4,000× magnification. Micrographs were taken at 25 kV in high vacuum mode.

### Sodium dodecyl sulfate-polyacrylamide gel electrophoresis (SDS-PAGE)

Soluble proteins in MOLM and FMOLM were determined as described previously, with minor modifications (Faurobert, 1997). Each 0.1 g sample was suspended in 1.5 mL Tris-HCl buffer (20 mM, pH 7.6) with 0.1% SDS, 5 mM dithiothreitol, and 5 µg/ mL protease inhibitor. The homogenized samples were centrifuged at 10,000× g for 15 min at 4 °C (5804R; Eppendorf, Hamburg, Germany) and the supernatants were transferred to Eppendorf tubes. The protein concentration for each sample was determined using a BCA Protein Assay kit (Pierce, Rockford, IL, USA). Approximately 20 µg protein was loaded onto a 12% SDS-PAGE gel and separated at 150 V for 60 min. After electrophoresis, the gel was stained with Coomassie Brilliant Blue R250 (Bio-Rad, Hercules, CA, USA) for 45 min and destained with 7% acetic acid.

### Statistical analysis

The general linear model (GLM) is appropriate for continuous variables. Moreover, the advantages are its simplicity, the ability to easily design and adjust the results for multiple factor. Then, the data were analyzed by one-way analysis of variance (ANOVA) using the general linear model (GLM) procedure of SAS software ([version] SAS Institute, Cary, NC, USA). The differences in the mean values were examined following a Least Significant Difference (LSD) test. A *p*-value of 0.05 was taken to indicate statistical significance. The results are expressed as means and standard deviation.

## Results

### Effect of inoculation order on the TP yield of FMOML

**Figure 1:**
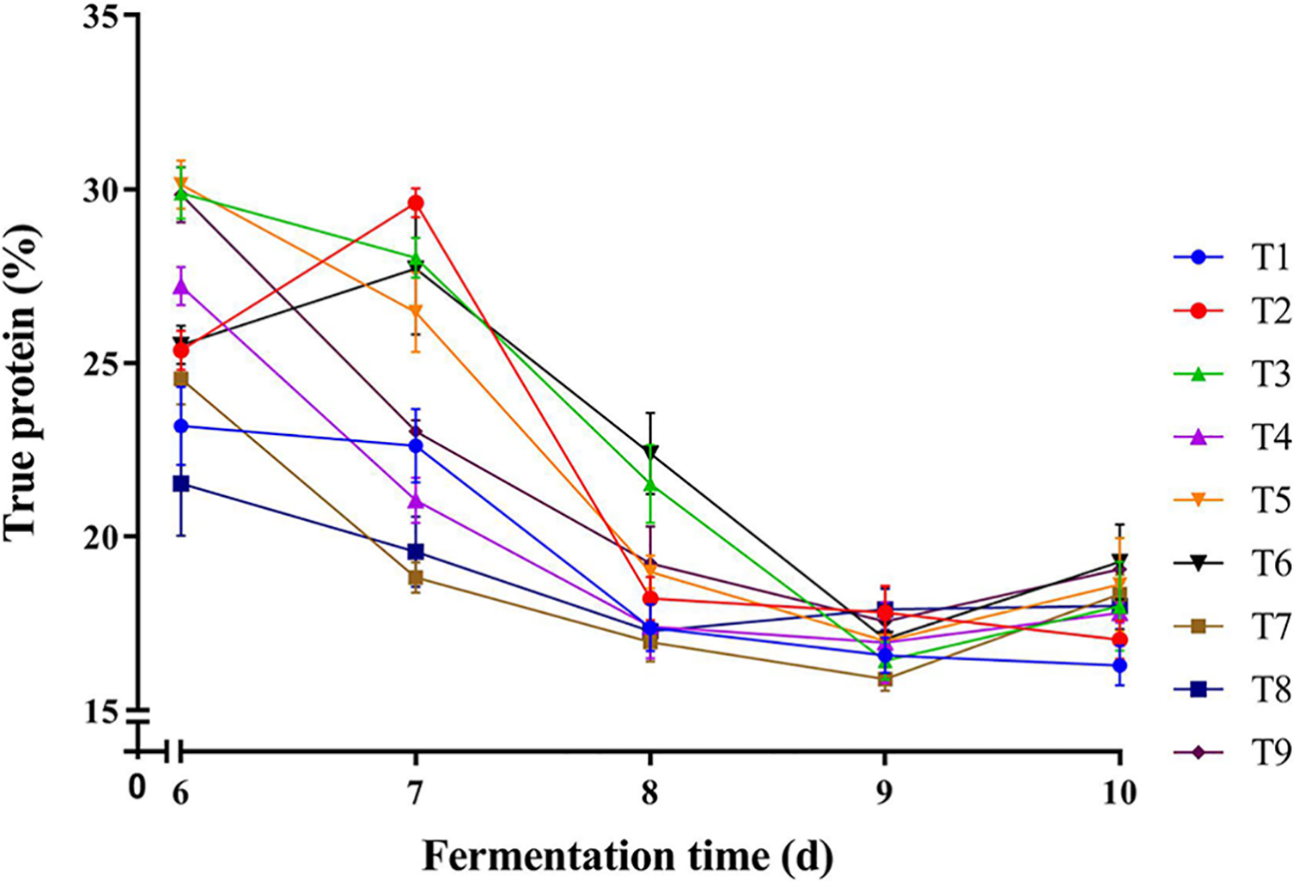
Effect of inoculation orders on TP yield of FMOLM.

To study the effect of the order of microorganism inoculation on the TP yield, MOLM was inoculated with *A. niger*, *C. utilis* and *B. subtilis* using nine different treatment orders. Samples were obtained from Day 6 over a 5-day period. As shown in Fig. 1, the maximum TP content in FMOLM (30.13%) was obtained on Day 6 with inoculation order T5. TP content gradually decreased after Day 6 for all inoculation orders, except T2 and T6. The maximum TP contents for T2 and T6 (29.61% and 27.71%, respectively) were obtained on Day 7; further incubation revealed a decline in TP yield. Inoculation order T5 and a 6-day fermentation cycle were selected for subsequent studies.

### Effect of various process parameters on the TP yield of FMOML

**Figure 2:**
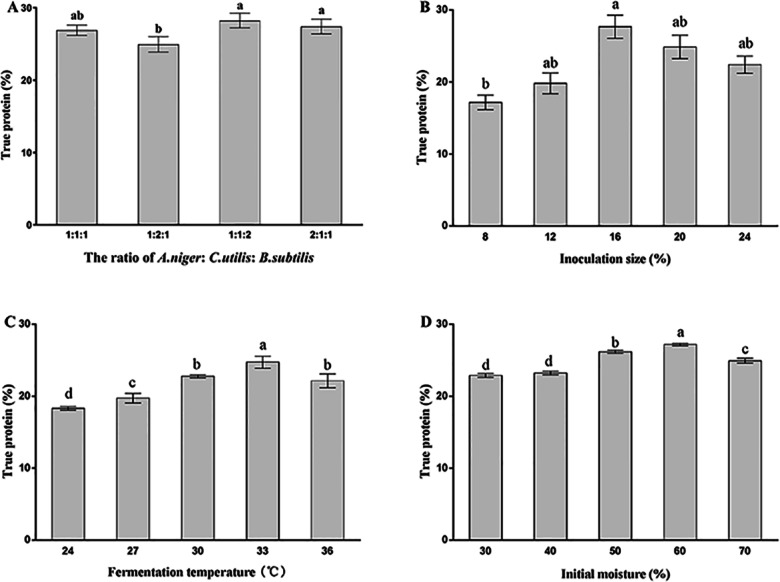
Effect of inoculation ratio (A), inoculation size (B), fermentation temperature (C), and initial moisture (D) on TP yield of FMOLM. Each parameter was tested at least in triplicate. Bars with different letters differed significantly (*P* < 0.05).

To determine the effects of inoculum ratio on MOLM protein enrichment, four ratios of *A. niger*: *C. utilis*: *B. subtilis* (1:1:1, 1:2:1, 1:1:2 and 2:1:1) were used (Fig. 2A). The TP content of FMOLM inoculated with a 1:2:1 ratio was significantly lower (*P* < 0.05) than that inoculated with a 1:1:2 or 2:1:1 ratio. No significant difference in TP content was observed between inoculum ratios of 1:1:2 (TP = 28.23%) and 2:1:1 (TP = 27.41%); therefore, an inoculum ratio of 1:1:2 was selected for downstream experiments.

The influence of inoculation size on growth kinetics and enzymatic production is illustrated in Fig. 2B. The TP content of FMOLM was highest at an inoculation size of 16% (27.67%), after which TP yield began to decline. A 16% inoculation size was therefore selected for downstream experiments.

Figure 2C shows the effects of fermentation temperature on TP production; the TP yield was significantly higher (*P* < 0.05) when SSF was performed at 33 °C compared to the other temperatures examined.

As shown in Fig. 2D, maximum TP content (27.19%) was attained when the initial moisture level was 60%. This is likely due to moisture interfering with the physical properties of substrate particles; high moisture levels reduced the porosity of the substrate, thus limiting oxygen exchange, while low moisture levels can reduce nutrient solubility, which in turn reduces microbial growth rates. Therefore, an initial moisture level of 60% was selected to optimize protein enrichment.

### Optimization of technical parameters by RSM and effects on TP yield

Table 3 shows the results of our BBD analysis. Regression analysis was performed using Design Expert 8.0.6 software (Stat-Ease Inc., Minneapolis, MN, USA) and the following quadratic equations:

**Table 3:** Box-Behnken design and the response for the TP content of FMOLM.

Run	A	B	C	Y
	Inoculation size (%)	Temperature (°C)	Time (d)	TP content (%)
1	1	0	−1	26.68
2	0	0	0	29.79
3	1	1	0	25.79
4	−1	−1	0	26.26
5	1	−1	0	27.72
6	1	0	1	24.73
7	−1	0	1	22.06
8	0	−1	1	24.63
9	0	0	0	30.06
10	−1	0	−1	27.77
11	0	−1	−1	25.74
12	0	1	−1	27.17
13	0	0	0	30.34
14	0	0	0	29.98
15	−1	1	0	23.43
16	0	1	1	22.42
17	0	0	0	31.05

*Y* = 30.24 + 0.68*A* − 0.69*B* − 1.69*C* + 0.22*AB* + 0.94*AC* − 0.91*BC*−2.06*A*^2^ − 2.38*B*^2^ − 2.87*C*^2^

where *Y* is TP yield, and *A*, *B*, and *C* are inoculation size, fermentation temperature, and fermentation time, respectively.

The ANOVA results are listed in Table 4. The F-value (23.97) indicated that the regression model was significant (*P* < 0.05). The lack-of-fit test was not significant (*P* = 0.1051), i.e., the regression model showed a good fit. The *R*^2^ correlation coefficient was 0.9686, and the corrected coefficient of determination (R_Adj_^2^) was 0.9282; thus, almost 93% of the variance in TP yield could be explained by the model. These analyses demonstrated good correlations between the experimental and predicted values. ANOVA showed that inoculation size and fermentation temperature significantly affected TP yield (*P* < 0.05), while fermentation time had a highly significant effect (*P* < 0.01).

**Table 4:** Analysis of variance for the response surface quadratic model.

Source	Sum of squares	DF	Mean square	*F* value	*P* value
Model	122.67	9	13.63	23.97	0.0002^**^
A	3.65	1	3.65	6.41	0.0391^*^
B	3.84	1	3.84	6.75	0.0356^*^
C	22.85	1	22.85	40.18	0.0004^**^
AB	0.20	1	0.20	0.36	0.5695
AC	3.53	1	3.53	6.22	0.0414^*^
BC	3.31	1	3.31	5.82	0.0465^*^
A^2^	17.90	1	17.90	31.48	0.0008^**^
B^2^	23.89	1	23.89	42.01	0.0003^**^
C^2^	34.73	1	34.73	61.07	0.0001^**^
Residual	3.98	7	0.57		
Lack of fit	3.01	3	1.00	4.15	0.1051
Pure error	0.97	4	0.24		
Cor total	126.65	16			

**Notes.**

DF degree of freedom Cor total totals of all information corrected for the mean

* Significant at *P* < 0.05.

** Highly significant at *P* < 0.01.

**Figure 3:**
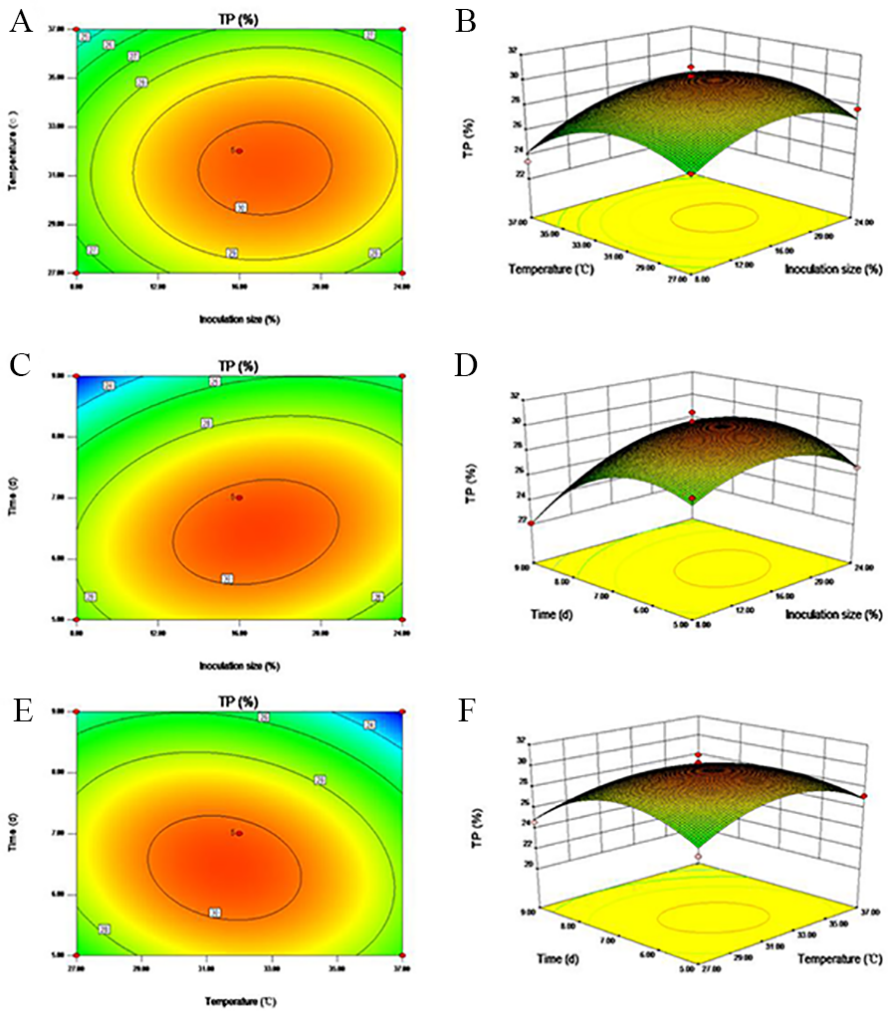
Three dimensional (3-D) surface graphs and Contour plots illustrating the interaction of (A–B) inoculation size and temperature, (C–D) inoculation size and time, and (E–F) temperature and time on TP yield.

Response surface plots and contour plots can be sued to visualize the interrelationships of independent variables when seeking to optimize conditions. Figure 3 shows the significant interactions between inoculation size and time, and temperature and time (*P* < 0.05), but no significant interaction was observed between inoculation size and temperature. The BBD analysis predicted that the following conditions would achieve the optimal TP yield: inoculation size, 23.84%; fermentation temperature, 31.63 °C; and fermentation time, 6.76 days. The estimated TP yield was 28.99% under these optimized conditions.

For convenience, the total inoculation size was adjusted to 24%, the temperature to 32 °C, and the time to 6.5 days. The other optimized process parameters were as follows: *C. utilis* and *B. subtilis* were inoculated 24 h later than *A. niger*, the initial moisture level was set to 60%, and the inoculum ratio (*A. niger*: *C. utilis*: *B. subtilis*) was 1:1:2. Under these conditions, the FMOLM TP content was 28.37%, demonstrating the applicability of the model for SSF optimization. Notably, the TP content of unfermented MOLM was 18.78%, demonstrating an increase in TP yield of 33.80% through the application of SSF.

### Changes in chemical composition during SSF

The nutrient contents of MOLM before and after optimized SSF processing are presented in Table 5. The FMOLM had increased levels of CP, TCP-SP, small peptides, NDF, ash, Ca, total P and hemicellulose compared to MOLM. The contents of CP, small peptides and ash in FMOML were 30.19%, 6.44%, and 10.13%, respectively, representing significant increases (*P* < 0.05) of 42.95%, 21.74%, and 25.84%, respectively, compared to MOLM. The concentrations of CF, total sugar, reducing sugar, tannin and phytic acid were significantly lower (*P* < 0.05) in FMOLM compared to MOLM, with respective reductions of 7.97%, 46.38%, 78.41%, 57.14% and 39.23%. Moreover, the total AAs, including dispensable and indispensable AAs, also significantly increased (*P* < 0.05) after fermentation. With the exception of lysine, all detectable AAs were enhanced in FMOLM. Notably, the total AA level increased by 16.42% in FMOLM. The IVPD was 65.05% in FMOLM, which represented a significant increase (51.77%; *P* < 0.05) relative to MOLM.

### Sodium dodecyl sulfate–polyacrylamide gel electrophoresis

**Figure 4:**
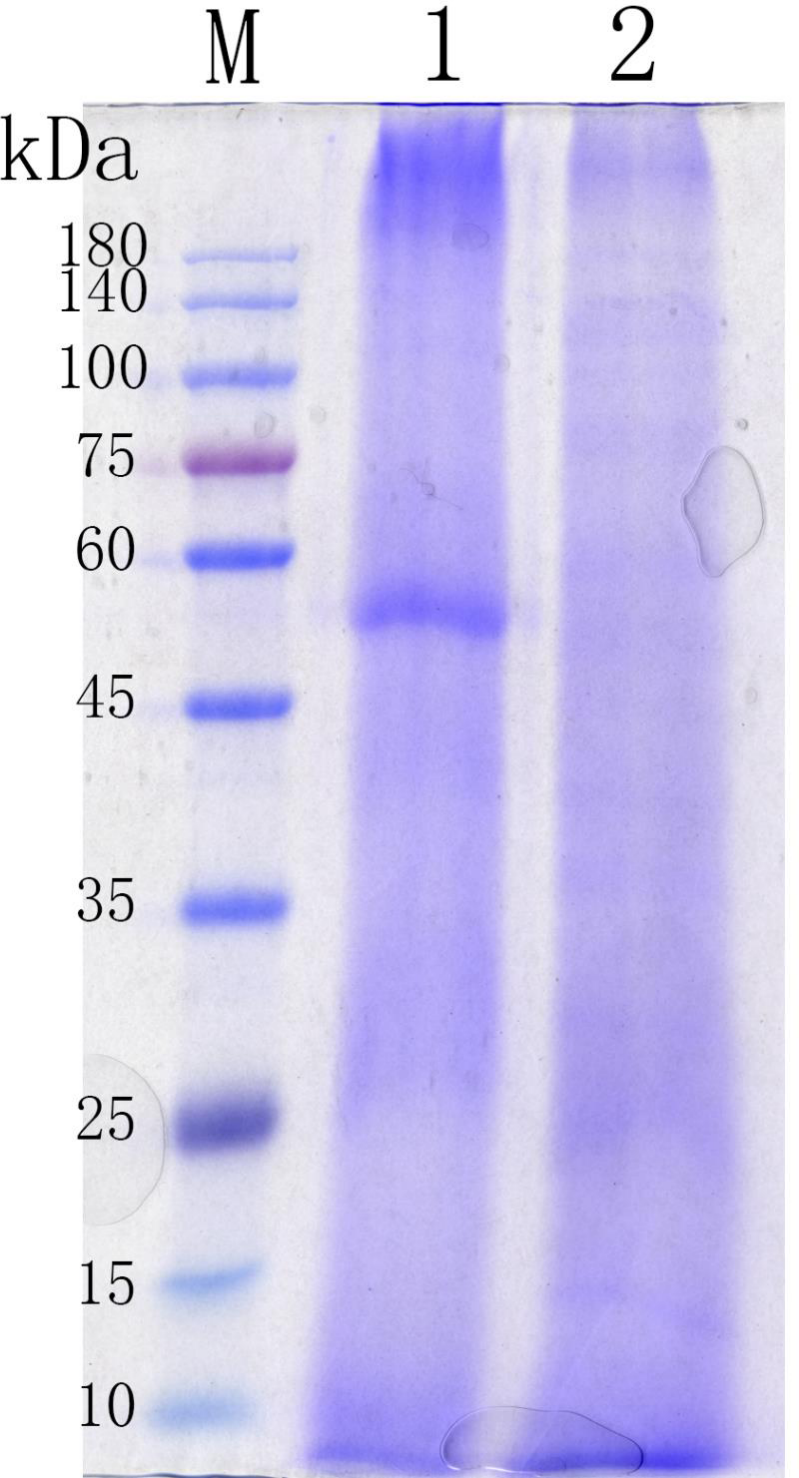
SDS-PAGE analysis of protein profiles in *Moringa oleifera* leaf meal (MOLM) before and after fermentation. Lane M: protein MW markers (10–180 kDa); Lane 1: *Moringa oleifera* leaf meal (MOLM); Lane 2: Fermented *Moringa oleifera* leaf meal (FMOLM).

As shown in Fig. 4, the molecular weight of the main protein fraction extracted from MOLM was approximately 55 kDa; however, this large-sized protein band was almost completely removed after MOLM fermentation. FMOLM protein bands were instead predominantly observed in the low molecular weight region, at around 15 kDa.

### Microstructure of MOLM and FMOLM

**Figure 5:**
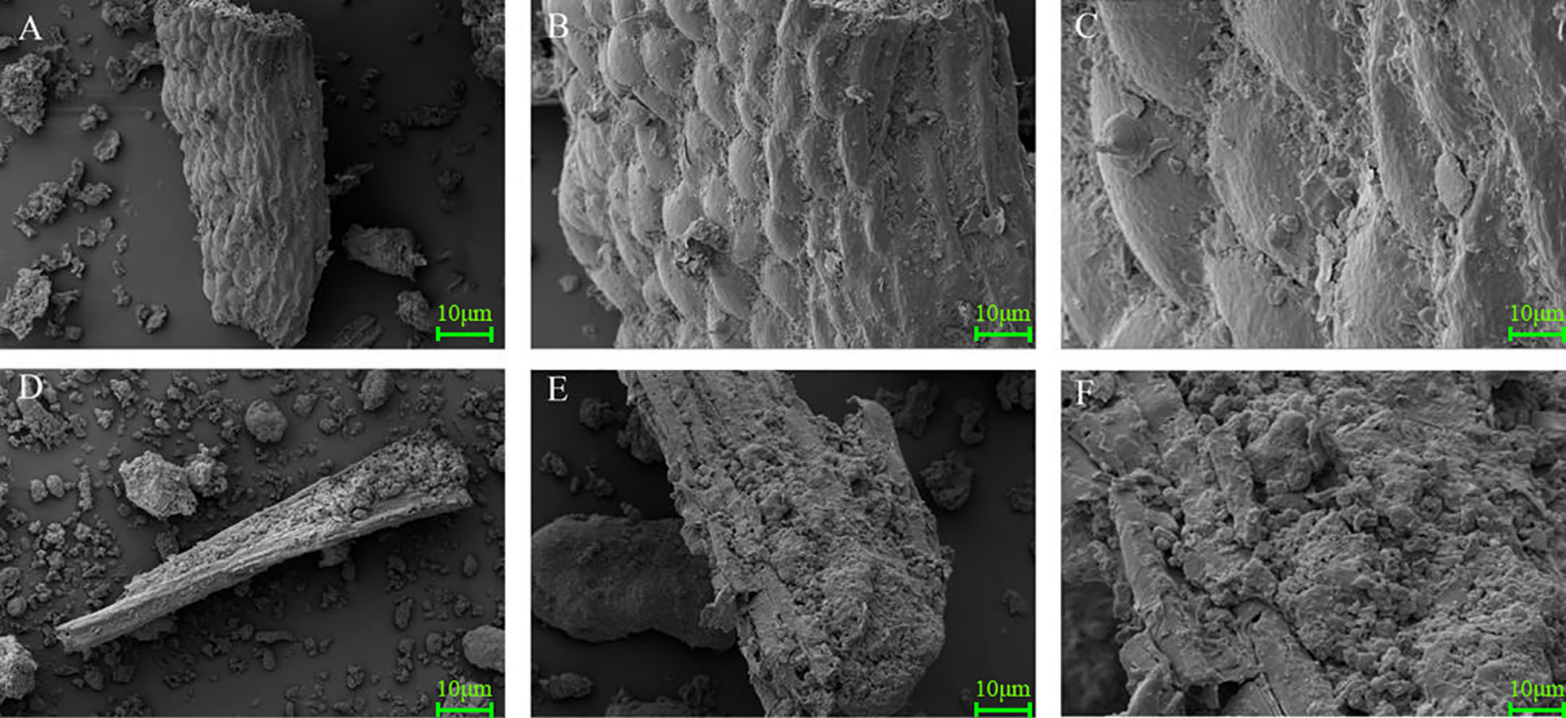
Scanning electron microscope images (×500, ×1,500, ×4,000) of MOLM (A, B, and C) and FMOLM (D, E, and F).

Figure 5 shows the microstructures of MOLM and FMOLM obtained using SEM at 500-, 1,500- and 4,000-fold magnification. At 500-fold magnification, unfermented MOLM displayed a relatively complete structure with a smooth and regular surface, whereas an irregular shape and rough surface were mainly observed in FMOLM. At 1,500-fold magnification, the lignocellulose structure of MOLM was intact; the FMOLM lignocellulose structure was more fragmented, with visible holes therein. At 4,000-fold magnification, numerous microorganisms appear to be attached to the FMOLM exterior, and surface structure of lignocellulose seemed to be partially broken.

**Table 5:** Nutrient composition of fermented *Moringa oleifera* leaf meal (FMOLM) and unfermented *Moringa oleifera* leaf meal (MOLM), as DM basis.

Item	MOLM	FMOLM
CP, %	21.12 ± 0.89^b^	30.19 ± 1.11^a^
TCP-SP, %	5.40 ± 0.04^b^	6.55 ± 0.03^a^
Small peptide, %	5.29 ± 0.01^b^	6.34 ± 0.04^a^
NDF, %	51.62 ± 2.79^b^	57.32 ± 1.72^a^
ADF, %	37.93 ± 2.01	34.95 ± 5.26
Hemicellulose, %	13.57 ± 2.63^b^	22.15 ± 1.34^a^
Cellulose, %	25.37 ± 4.09	21.50 ± 2.39
Lignin, %	10.44 ± 2.02	10.55 ± 2.27
CF, %	27.35 ± 1.14^a^	25.17 ± 1.05^b^
EE, %	4.68 ± 0.11	4.42 ± 0.23
Ash, %	8.05 ± 0.44^b^	10.13 ± 0.32^a^
Ca, %	0.90 ± 0.01	1.14 ± 0.11
P, %	0.22 ± 0.03	0.29 ± 0.01
IVPD, %	42.86 ± 0.23^b^	65.05 ± 0.89^a^
Total sugar, %	13.41 ± 1.92^a^	7.19 ± 1.30^b^
Reducing sugar, %	0.88 ± 0.20^a^	0.19 ± 0.12^b^
Tannin, mg/g	8.39 ± 1.19^a^	3.63 ± 0.71^b^
Phytic acid, %	5.94 ± 0.07^a^	3.61 ± 0.01^b^
Indispensable AA, %	
Arg	0.70 ± 0.10	0.75 ± 0.07
His	0.24 ± 0.05^b^	0.29 ± 0.08^a^
Ile	0.49 ± 0.09^b^	0.65 ± 0.07^a^
Leu	1.14 ± 0.22	1.38 ± 0.28
Lys	0.66 ± 0.10	0.59 ± 0.13
Phe	0.69 ± 0.18	0.85 ± 0.25
Thr	0.67 ± 0.10^b^	0.81 ± 0.17^a^
Val	0.67 ± 0.10^b^	0.86 ± 0.10^a^
Trp	0.33 ± 0.15^b^	0.43 ± 0.13^a^
Dispensable AA, %	
Ala	0.92 ± 0.06	1.07 ± 0.13
Asp	1.39 ± 0.15^b^	1.62 ± 0.26^a^
Cys	0.07 ± 0.01^b^	0.08 ± 0.02^a^
Glu	1.67 ± 0.42	1.85 ± 0.57
Gly	0.78 ± 0.16	0.85 ± 0.34
Pro	0.65 ± 0.03	0.78 ± 0.02
Ser	0.68 ± 0.11	0.82 ± 0.20
Tyr	0.37 ± 0.09^b^	0.45 ± 0.12^a^
Total AA	12.12 ± 1.94^b^	14.11 ± 2.28^a^

**Notes.**

Values are means of three replicates per treatment.

Different superscript letters indicate significate differences at *P* < 0.05 in the row.

## Discussion

Recent evidence suggests that several important biochemical processes are impeded if only a single strain is used for SSF, and that co-cultures are necessary to achieve optimal SSF outcomes (Dhiman et al., 2015; Wang, Bai & Liang, 2006; Xie et al., 2016). Several fungi, yeasts, and bacterial strains are able to grow synergistically, and are widely applied as co-cultures to improve the nutritional quality of unconventional feed resources (Yao et al., 2018). As reported by Wang et al. (2018b), the nutritional quality and digestibility of MOLM was significantly improved via SSF using *A. niger*. The application of multi-strain mixed fermentation is relatively unexplored, and SSF of MOLM using a *A. niger*, *C. utilis* and *B. subtilis* co-culture has not yet been reported. In the present study, these microorganisms were combined to ferment MOLM, which not only reduced the content of anti-nutritional compounds, but also increased the protein content. Previous reports indicated that co-culture was superior to a single strain, which was attributed to the use of all available substrates during multi-strain fermentation (Del Fresno et al., 2017; Yadav et al., 2014). The success of MOLM fermentation can be explained in part by the fact that *A. niger* has greater cellulose, pectinase, and amylase activities; this facilitates the degradation of cellulose and starch, which in turn increases the available carbon. *C. utilis* and *B. subtilis* can also convert non-protein nitrogen into microbial protein, and secrete enzymes that accelerate cellulose and starch degradation, thereby promoting the mycelial growth of *A. niger*. *B. subtilis* can also produce additional functional compounds, thus improving the palatability of MOLM for livestock.

In the early stages of fermentation, microorganisms compete with each other for nutrients. The growth rate of *B. subtilis* is initially faster than those of *A. niger* and *C. utilis*, which decreases nutrient availability and limits the growth rates of fungal species. This is consistent with a study by Jia et al. (2019), who found that inoculating *A. niger* first facilitated enzymatic hydrolysis, which increased the pool of available carbon and allowed the growth and reproduction of other microbes*.* The selection and optimization of process parameters such as inoculation size, initial moisture content, fermentation temperature, and fermentation length are also important to improve the efficiency of SSF (Abu Yazid et al., 2017). When the inoculation size is too small, the microbes are unable to reach sustainable culture densities, rendering the substrate susceptible to contamination and feed spoilage. On the other hand, large inoculation sizes result rapid in nutrient depletion, and cause a crowding effect that limits the growth of microorganisms as well as the synthesis of microbial protein (Xu et al., 2018). The initial moisture content also plays a pivotal role in protein enrichment during SSF (Orzua et al., 2009). Fungi and bacteria have different moisture content requirements. Generally, a moisture content of 30–60% is necessary to initiate fungal growth, whereas a minimum moisture content of 60% is required for the growth and conidiation of bacteria (Behera & Ray, 2016). A low moisture content reduces nutrient solubility, consequently limiting microbial growth. Conversely, a high moisture content can cause a decline in the porosity of the substrates and induces material adhesion, which in turn influences oxygen transfer and temperature (Sadh, Duhan & Duhan, 2018; Sousa et al., 2018).

High temperatures can cause denaturation of some proteins and enzymes. On the other hand, the positive influence of elevated temperature on protein yield can be explained by increased secretion of enzymes by certain microorganisms (Abraham, Gea & Sanchez, 2014; Yazid, Barrena & Sanchez, 2016). Suboptimal fermentation times can also negatively affect the growth of microorganisms (Ezeilo et al., 2019). In this study, protein enrichment was reduced as the fermentation time increased due to the accumulation of metabolic waste and overconsumption of substrates. RSM appears to be a satisfactory and effective tool for optimizing process parameters when compared to classical statistical methods. Zuo et al. (2018) reported that the TP content of fermented sweet potato beverage residues mixed with peanut shells increased 2- to 3-fold compared to unfermented substrates under RSM-optimized conditions. In the present study, SSF of MOLM with mixed strains resulted in a 33.80% increase in the TP content through parameter optimization by RSM.

Furthermore, FMOLM contained more CP, TAA and small peptides, which accords with the results obtained with fermentation of soy meal (Chen, Madl & Vadlani, 2013), rapeseed meal (Hao et al., 2020), and corn-soybean meal (Shi et al., 2017). An increase in CP levels following FMOLM can be ascribed to the loss of DM (mainly carbohydrates) and the synthesis of fungal biomass proteins (Hong, Lee & Kim, 2004). The AA content of FMOLM increased by about 16.42% relative to unfermented MOLM in our study. (Valdivienavarro et al., 2020) reported higher AA content of MOLM than alfalfa forage meal. In this study, the TCP-SP comprised small peptides and FAAs, which increased due to the digestion of macromolecular proteins and the high levels of proteases secreted by *A. niger*, *C. utilis,* and *B. subtilis* during fermentation. SDS-PAGE analysis showed that protein bands around 55 kDa in size are predominant in MOLM, consistent with the findings of Wang et al. (2016); however, large-sized protein bands were not observed after fermentation. SSF is an important element of feedstuff processing to enhance protein digestion (Wang et al., 2018a). In the present study, the IVPD of FMOLM was significantly higher than that of MOLM. SEM analysis indicated that the higher digestibility of FMOLM may be due to degradation of the structure of lignocellulose biomass by SSF, thereby increasing the accessibility of nutrients.

A combination of fungi and bacteria could produce a highly active and complete cellulase system. In fact, *A. niger* and *B. subtilis* have demonstrated multi-carbohydrase, phytase, and tannase activities (Medeiros et al., 2018). The decrease in CF and total sugar content in this study suggested that cellulose was broken down into monosaccharides. It is likely that *C. utilis* utilized these simple sugars to proliferate, thereby avoiding the suppressive effects of metabolites. Phytic acid and tannin are the main anti-nutritional factors in MOLM (Chodur et al., 2018; Ogbe & Affiku, 2020). High levels of tannin have an adverse effect on animal productivity and digestibility, as well as palatability (Huang et al., 2017). Likewise, the phytic acid of MOLM cannot be absorbed by monogastric animals, and can interfere with mineral availability (Singh & Satyanarayana, 2011). The presence of phytase and tannase, which are produced by bacteria and fungi during SSF, can degrade these adverse substances. Commonly, *Aspergillus* is considered to be the primary source of these enzymes. Sandhya, Sridevi & Suvarnalathadevi (2019) found that phytase purified from *A. niger* S2 was effective in degrading phytic acid in grass and hay. Previous studies also reported that many extracellular degradation enzymes secreted by *A. niger*, such as lignocellulosic hydrolyzing enzymes, phytase, and tannase, were detected during fermentation (Liu et al., 2018; Shi et al., 2016). Phytase and tannase have also been shown to be produced by *B. subtilis* during SSF (Banerjee, Mondal & Pati, 2001; Yoon et al., 1996). In our study, the contents of tannin and phytic acid markedly decreased after fermentation, possibly due to enzymatic hydrolysis. The alterations in chemical composition can hence be linked to the enzymes produced by microorganisms. To further explore the degradation mechanisms of SSF, the composition of extracellular enzymes secreted by microorganisms during fermentation should be investigated in future studies.

## Conclusions

In this work, SSF of MOLM by a mixed-strain culture comprising *A. niger*, *C. utilis,* and *B. subtilis* was optimized for maximum TP content using RSM. The optimal fermentation conditions were as follows: ratio of *A. niger*, *C. utilis* and *B. subtilis* of 1:1:2; *A. niger* inoculated 24 h prior to *C. utilis* and *B. subtilis* inoculation; total inoculation size of 24%; temperature of 32 °C; fermentation time of 6.5 d; and initial water content of 60%. Under these conditions, the TP content of FMOLM reached 28.37%, which is 33.80% higher than the content in pre-optimized FMOLM. Furthermore, the results showed an increase in CP, AA and small peptides, as well as a reduction in CF, total sugar, and anti-nutritional factors during fermentation of MOLM. Microscopy revealed that the surface microstructure of the MOLM was disrupted, and the degradation of large-size protein bands was observed by SDS-PAGE. The IVPD of MOLM was also remarkably improved by SSF. Therefore, our results suggest that SSF is conducive to improving the nutritional value of MOLM, demonstrating its potential value for the livestock industry.

##  Supplemental Information

10.7717/peerj.10358/supp-1 Supplemental Information 1 Moringa oleifera leaf meal before after fermentation

10.7717/peerj.10358/supp-2 Supplemental Information 2 Statistical analysis

10.7717/peerj.10358/supp-3 Supplemental Information 3 Raw data

## Additional Information and Declarations

### Data Availability

The following information was supplied regarding data availability: The raw measurements are available in the Supplemental Files.
