# Supplementary figures and images for: Solid state fermentation of Moringa oleifera leaf meal by mixed strains for the protein enrichment and the improvement of nutritional value
Source: PeerJ. 2020 Nov 18;8:e10358. doi: 10.7717/peerj.10358 (PMC7680055; doi:10.7717/peerj.10358)

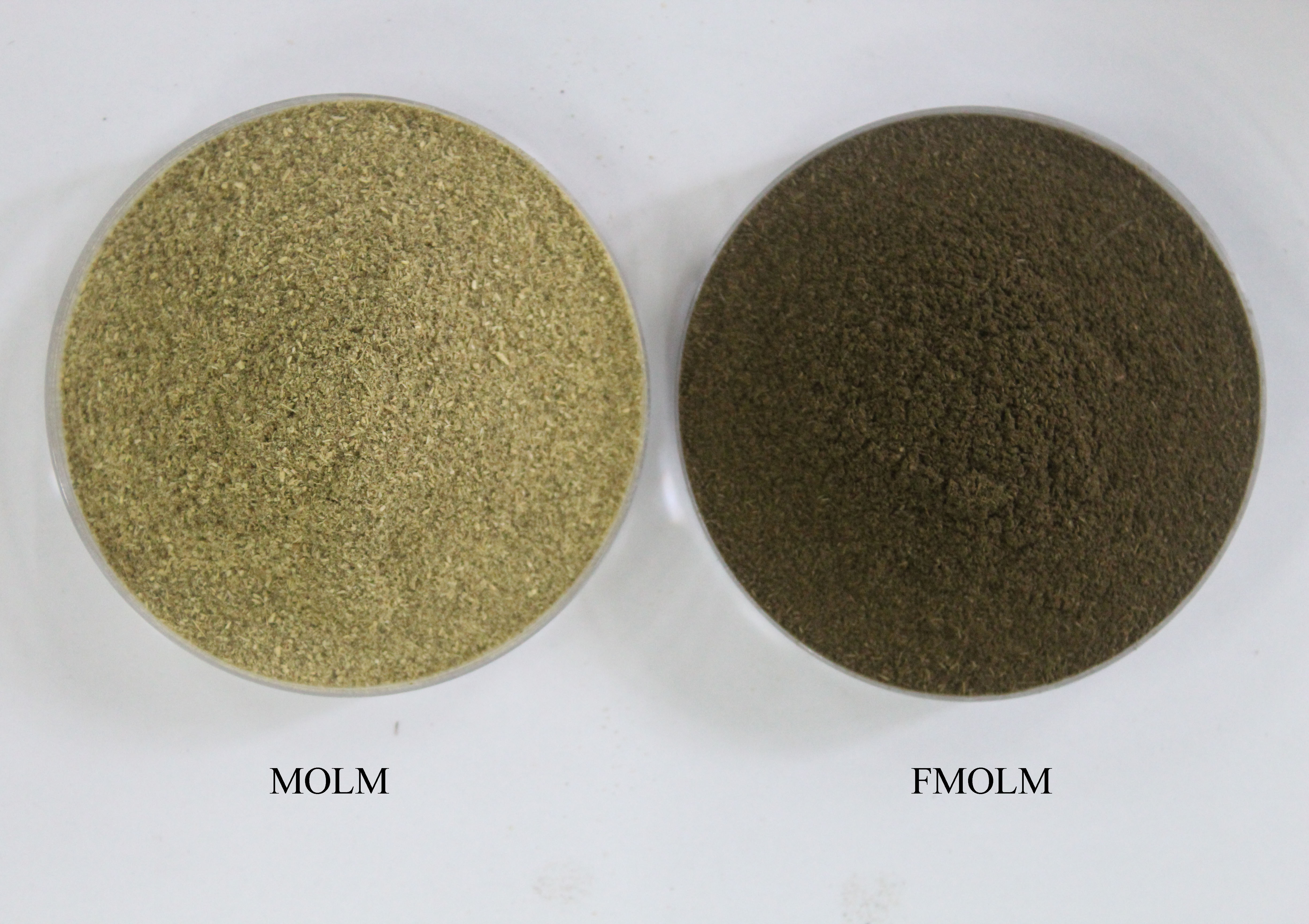

Supplement: Supplemental Information 1 [file peerj-08-10358-s001.jpg]
